# Monophasic-quadri-burst stimulation robustly activates bilateral swallowing motor cortices

**DOI:** 10.3389/fnins.2023.1163779

**Published:** 2023-05-25

**Authors:** Minoru Fujiki, Nobuhiro Hata, Mitsuhiro Anan, Wataru Matsushita, Yukari Kawasaki, Hirotaka Fudaba

**Affiliations:** Department of Neurosurgery, School of Medicine, Oita University, Oita, Japan

**Keywords:** swallowing dysfunction, magnetic stimulation, quadripulse theta-burst stimulation, motor evoked potentials, quadripulse stimulation

## Abstract

A stable, reliable, non-invasive, quantitative assessment of swallowing function remains to be established. Transcranial magnetic stimulation (TMS) is commonly used to aid in the diagnosis of dysphagia. Most diagnostic applications involve single-pulse TMS and motor evoked potential (MEP) recordings, the use of which is not clinically suitable in patients with severe dysphagia given the large variability in MEPs measured from the muscles involved in swallowing. Previously, we developed a TMS device that can deliver quadripulse theta-burst stimulation in 16 monophasic magnetic pulses through a single coil, enabling the measurement of MEPs related to hand function. We applied a system for MEP conditioning that relies on a 5 ms interval-monophasic quadripulse magnetic stimulation (QPS5) paradigm to produce 5 ms interval-four sets of four burst trains; quadri-burst stimulation (QBS5), which is expected to induce long-term potentiation (LTP) in the stroke patient motor cortex. Our analysis indicated that QBS5 conditioned left motor cortex induced robust facilitation in the bilateral mylohyoid MEPs. Swallowing dysfunction scores after intracerebral hemorrhage were significantly correlated with QBS5 conditioned-MEP parameters, including resting motor threshold and amplitude. The degree of bilateral mylohyoid MEP facilitation after left side motor cortical QBS5 conditioning and the grade of severity of swallowing dysfunction exhibited a significant linear correlation (*r* = −0.48/−0.46 and 0.83/0.83; *R*^2^ = 0.23/0.21 and 0.68/0.68, *P* < 0.001; Rt./Lt. side MEP-RMT and amplitudes, respectively). The present results indicate that RMT and amplitude of bilateral mylohyoid-MEPs after left motor cortical QBS5 conditioning as surrogate quantitative biomarkers for swallowing dysfunction after ICH. Thus, the safety and limitations of QBS5 conditioned-MEPs in this population should be further explored.

## 1. Introduction

Swallowing is a complex physiological phenomenon consisting of voluntary movements and reflexes, which are accomplished by the perfectly coordinated action of 50 pairs of related muscle groups innervated by the bilateral hemispheres. In older adults, there is a loss of oral and pharyngeal mucosal sensation and a general weakening of the pharyngeal muscle groups, which delays laryngeal elevation in response to the entry of food mass into the pharynx, resulting in aspiration (Macrae et al., 2014). Most cases of pneumonia in older adults are aspiration pneumonia, which is a major cause of death among both older adults and patients with stroke. Despite the high risk of aspiration pneumonia after stroke, stable and reliable methods for the non-invasive quantification of swallowing function remain to be fully established. Although the popularity of non-invasive transcranial magnetic stimulation (TMS) for assessing swallowing function has grown; motor evoked potentials (MEPs) recorded from the swallowing muscles are problematic because of the large standard deviation in their values (Macrae et al., 2014). To address this issue, we developed a system for measuring MEPs that relies on monophasic quadripulse theta-burst magnetic stimulation (QTS). In this system, the outputs of 16 stimulators (**Magstim 200**^**2**^ is a product by the Magstim Co., Ltd.) are combined using a specially designed module, following which four sets of four-monophasic magnetic pulses are transmitted via a single coil (Fujiki et al., 2022; patent number 7189594). We applied a system for MEP conditioning that relies on a 5 ms interval-monophasic quadripulse magnetic stimulation (QPS5) paradigm (Hamada et al., 2008), which is known to induce long-term potentiation (LTP) in the human hand motor cortex. Furthermore, Tsutsumi et al. (2014) revealed that QPS5, well established method for motor cortical LTP induction using four monophasic pulses induces bilateral motor cortical facilitaion in transcallosal interhemispheric and intracortical facilitation mechanisms. Furthermore, a recent study showed that the swallowing motor cortex in normal subjects has a right hemispheric dominance of lateralization, and electro acupuncture instantly promotes excitability in the bilateral swallowing motor cortex (Tang et al., 2022). In the present study, we aimed to investigate whether 5 ms interval-four burst trains; quadri-burst stimulation (QBS5) can also strongly amplify swallowing-related MEPs and determine whether the quantifiable functional parameters obtained during TMS-based assessments exhibit correlations with neurogenic swallowing disorders.

## 2. Materials and methods

### 2.1. Participants

We analyzed data from the same 65 participants included in our previous study, which demonstrated the superiority of monophasic quadripulse theta-burst magnetic stimulation (QTS) in patients with hand motor paralysis following left hemisphere intracerebral hemorrhage (ICH) (Fujiki et al., 2022). The original study included a control group of 10 healthy, right-handed men (age range: 40–68 years; mean ± SD: 58.5 ± 10.8 years) without any contraindications to TMS, as well as 65 patients with hypertension who had experienced putaminal ICH (5 women, 60 men; age range: 55–80 years; mean ± SD: 68.9 ± 11.8 years). No individuals in the control group took regular medications, and none had a history of psychiatric or neurologic illness (Rossi et al., 2009). The original study was approved by the ethics committee of the School of Medicine at Oita University (protocol number: 265), and all individuals in the control and patient groups provided written informed consent to participate.

The participants, 65 consecutive patients, who underwent conservative treatment without surgery between January 2008 and December 2021, demonstrated impaired motor function, which was mostly caused by the compression or destruction of the corticospinal tract due to hemorrhage (>5 and <30 ml in volume, symptom onset <24 h before admission, clear consciousness, no neurological deficits apart from swallowing and motor dysfunction). The median time from onset to examination was 3.3 (range: 1–7) days. The severity of swallowing dysfunction was evaluated using a modified water swallowing test (MWST), in which 3-ml of cold water was placed on the floor of the mouth using a 5-ml syringe. The patient was instructed to swallow, and their swallowing was scored as follows: 1, inability to swallow accompanied by choking and/or changes in breathing; 2, ability to swallow but with changes in breathing; 3, ability to swallow without changes in breathing but with choking and/or wet hoarseness; 4, successfully swallowing without choking or wet hoarseness; 5, original score of 4 with additional deglutition (dry swallowing) more than twice within 30 s (Tohara et al., 2003). Patients who were unable to attempt the MWST were excluded (*n* = 10). The mean swallowing score (range: 1–5) was 2.55 ± 1.72 (score 1, *n* = 12; score 2, *n* = 11; score 3, *n* = 12; score 4, *n* = 10; score 5, *n* = 10). Patient characteristics are shown in Table 1.

**TABLE 1:** Summary of patient characteristic.

Case	Age	Sex	MWST grade	Type of ICH	Location	Hematoma volume (mL)	Days after onset
1	65	M	5	Subcortical	Frontal	18	2
2	80	M	5	Subcortical	Frontal	7	3
3	80	M	5	Subcortical	Frontal	12	3
4	78	M	5	Subcortical	Frontal	19	3
5	67	M	5	Subcortical	Frontal	10.4	5
6	66	M	5	Subcortical	Frontal	5	1
7	73	M	5	Subcortical	Frontal	8	2
8	58	M	5	Subcortical	Frontal	28	7
9	68	M	5	Subcortical	Parietal	19	7
10	60	M	5	Subcortical	Frontal	5	1
11	62	M	4	Subcortical	Temporal	12	1
12	64	M	4	Subcortical	Frontal	18	5
13	71	M	4	Subcortical	Diffuse	11	5
14	63	F	4	Subcortical	Frontal	12	4
15	66	F	4	Basal ganglia	Putamen	5	3
16	69	F	4	Subcortical	Frontal	11.1	5
17	66	F	4	Basal ganglia	Putamen	6	5
18	65	F	4	Subcortical	Frontal	11	3
19	64	M	4	Subcortical	Temporal	15	3
20	60	M	4	Subcortical	Parietal	5.4	3
21	68	M	3	Basal ganglia	Putamen	12	8
22	59	M	3	Basal ganglia	Putamen	11	7
23	55	M	3	Basal ganglia	Putamen	8	2
24	63	M	3	Subcortical	Parietal	17	5
25	62	M	3	Subcortical	Temporal	15.5	5
26	79	M	3	Subcortical	Frontal	11	5
27	80	M	3	Subcortical	Frontal	10	5
28	81	M	3	Subcortical	Temporal	10	2
29	80	M	3	Subcortical	Parietal	11	2
30	63	M	3	Basal ganglia	Putamen	12	2
31	60	M	3	Basal ganglia	Putamen	17	2
32	78	M	3	Subcortical	Parietal	15	2
33	63	M	2	Subcortical	Parietal	8.8	1
34	63	M	2	Subcortical	Frontal	9.8	1
35	80	M	2	Subcortical	Frontal	10.2	1
36	78	M	2	Subcortical	Frontal	8.2	3
37	80	M	2	Subcortical	Frontal	19	3
38	66	M	2	Subcortical	Frontal	11	3
39	69	M	2	Subcortical	Temporal	12	2
40	79	M	2	Basal ganglia	Putamen	5.5	2
41	68	M	2	Subcortical	Parietal	15	2
42	80	M	2	Basal ganglia	Putamen	6.1	1
43	65	M	2	Basal ganglia	Putamen	10	3
44	62	M	1	Basal ganglia	Putamen	5	3
45	61	M	1	Basal ganglia	Putamen	10	3
46	80	M	1	Subcortical	Parietal	8.7	3
47	71	M	1	Subcortical	Parietal	14.5	3
48	77	M	1	Basal ganglia	Putamen	6.5	3
49	68	M	1	Basal ganglia	Putamen	8.8	3
50	59	M	1	Subcortical	Frontal	19	3
51	80	M	1	Subcortical	Parietal	19	3
52	63	M	1	Basal ganglia	Putamen	5.2	3
53	68	M	1	Basal ganglia	Putamen	5.9	3
54	68	M	1	Basal ganglia	Putamen	8	3
55	64	M	1	Basal ganglia	Putamen	7	3

MEP, motor evoked potential; RMT, resting motor threshold; QBS5, quadr burst stimulation-5ms interburst interval; QPS, quadripulse stimulation; SP, single pulse; MWST, modified water swallowing test. Fifty five patients with swallowing dysfunction following left hemisphere intracerebral hemorrhage.

### 2.2. System configuration and control study

**FIGURE 1:**
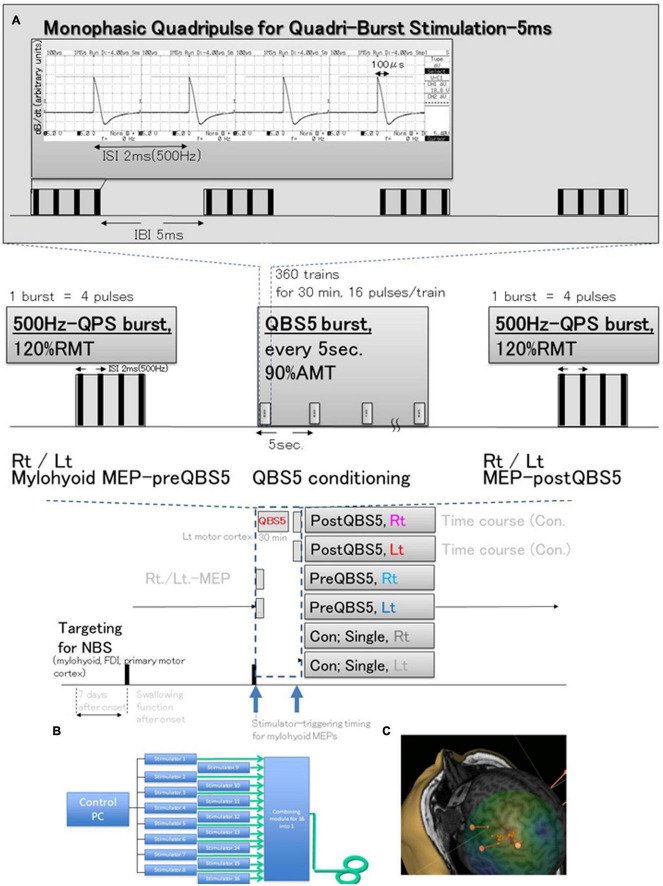
Schematic illustration of the experimental protocol and stimulus configurations. Comparison between standard single-pulse magnetic stimulation-induced-MEPs, QPS; single 500-Hz bursts, and before and after QBS-5; four 500-Hz bursts, repeated at 200-Hz (i.e., 5 ms interbust interval) stimulation MEPs. **(A)** Stimulus conditions: five-ms-quadri-burst stimulation [QBS5], 500-Hz quadripulse single train stimulation [QPS], and monophasic single-pulse stimulation. QBS5 consisted of four bursts, each consisting of four high-frequency monophasic pulses which were delivered at 500 Hz (i.e., 2 ms-ISI), repeated at 200 Hz (i.e., 5-ms interburst interval), and delivered every 5 s continuously for 30 min, resulting in a total of 360 trains, 16 pulses/train. QPS consisted of four high-frequency monophasic pulses delivered at 500 Hz (i.e., 2-ms interstimulus interval [ISI]). MEP acquisitions were performed from the final burst at stimulus intensity of 120% RMT. Mylohyoid motor cortices were conditioned with QBS5 for 30 min at stimulus intensity of 90% AMT. Monophasic magnetic flux data recorded using ossiloscope and pickup coil was originated from our previous publication (Fujiki et al., 2022). **(B)** SP, QPS, or QBS5 was applied to the hand and mylohyoid muscle area of the left motor cortex with a PA directed monophasic magnetic QBS-induced MEPs device system, including a set of 16 separate magnetic stimulators (Magstim, 2002; The Magstim Co., Ltd., Wales, UK) connected with a specially designed combining module (Fujiki et al., 2022; patent number 7189594). This device combines the outputs from the 16 stimulators to deliver a train of 16 monophasic magnetic pulses through a single coil. QBS5, QPS, or SP were applied to the hand and mylohyoid area of the left motor cortex with a PA-directed monophasic pulse. **(C)** MEPs were recorded under the targeted primary motor cortex for mylohyoid muscle and first dorsal interosseous (FDI) muscle using a navigated brain stimulation system within 7 days after onset. The screenshot depicts a representative control case with the area mapped to identify the motor optimal location (hotspot) in the target muscle. Each dot on the scalp can be visualized as orange balls, and the red arrow shows the current direction in the brain. The colors show the relative strength of the E-field (red, high E-field strength; blue, low E-field strength). The position feedback indicator (small window on the right for repeated constant stimulation) provides real-time feedback for surface location-enabled manual holding and reliable targeting. QBS5, five-ms-quadri-burst stimulation; QPS, quadripulse stimulation; PA, posterior-anterior; ISI, inter-stimulus interval; IBI, inter-burst interval; RMT, resting motor threshold; TMS, transcranial magnetic stimulation; MEP, motor evoked potential; NBS, navigated brain stimulation.

Control studies for healthy participants [under electroencephalographic (EEGs) monitoring for detection of subclinical abnormalities] were performed as described in our previous study (Figure 1B; Fujiki et al., 2022) to test three different configurations of the posterior- anterior directed induced current flow (i.e., monophasic single pulse [SP], 500 Hz [i.e., 2-ms interstimulus interval (ISI)-quadripulse single train stimulation (QPS)] and QBS5) were validated in both hemisphere in each modality. A 70-mm figure-8 coil was used to deliver magnetic pulses at 1.2 times the resting motor threshold (RMT) for MEP recording. A QBS5 at the left hemisphere [four sets of four monophasic pulses at a frequency of 500 Hz, repeated at 200 Hz; i.e., 5-ms interburst interval, with an inter-train interval of 5-s] at 0.9 times active motor threshold (AMT) for left motor cortical conditioning was delivered for 30 min. A navigated brain stimulation system (NBS; Nexstim eXima; Nexstim Ltd., Helsinki, Finland) was used to target the primary motor cortex for the mylohyoid muscle using the first dorsal interosseous muscle (FDI) as a positive control. The NBS is an optical tracking system for precise TMS tracking in real time real-time (see Figure 1C).

**FIGURE 2:**
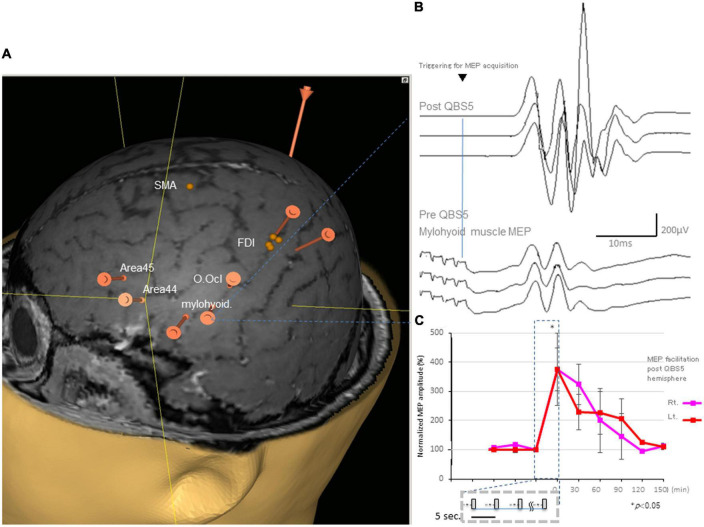
Representative MEPs following QPS recorded from the mylohyoid muscle and facilitation after QBS5 in a healthy control. **(A)** Target areas registered before mylohyoid and FDI mapping using the navigated brain stimulation (NBS) system [orange bars; mylohyoid, FDI muscle and orbicuralis oculi (O. Ocl.)], supplementary motor area [SMA], Brodmann’s area 44, 45 [Area 44, Area 45] for reference, respectively. QBS5, five-ms-quadri-burst stimulation; MEP, motor evoked potential; FDI, first dorsal interosseous. **(B)** Representative MEPs following QPS before (lower traces) and after (upper traces) recorded from the mylohyoid muscle in a healthy control. **(C)** MEP facilitation in after left motor cortical QBS5 conditioning in a healthy participant. Amplitudes increased by 350–370% when compared with those for pre conditioned-MEPs. Note that mylohyoid MEPs recorded from bilateral side was facilitated following QBS5-left hemisphere-conditioning.

Our preliminary study revealed that SP- mylohyoid MEPs were always constantly recordable only in healthy control. Stable and reproducible 500Hz QPS-mylohyoid MEPs were employed, as these may reflect MWST scores. Before and after the QBS5 conditionings, MEPs were measured to determine the RMT, amplitude, and latency (dark blue dashed box, Figures 1A, 2). After determining the hot spot of the FDI muscle using NBS, individual MRI-based anatomical maps placed orthogonally to the central sulcus on the NBS were used as candidate targets to guide further assessment of the cortical physiology of mylohyoid muscle activations in the left M1. The mylohyoid muscle “hotspot” was further identified as the location of the “strongest” mylohyoid MEP amplitude evoked with the lowest-intensity motor threshold that elicited discernible MEPs. After the motor cortical point was registered on the reconstructed 3D MRI images, Euclidian distances between the mylohyoid and FDI were calculated via the NBS (mm). When appropriate stimulation was delivered to the representative areas of M1, MEPs could be observed from the respective mylohyoid and FDI hot spots (Hannula et al., 2005; Macrae et al., 2014; Li et al., 2020). In accordance with previous report for FDI-MEPs (Hamada et al., 2008), AMT for conditioning of mylohyoid motor hotspot was defined as the lowest intensity to evoke mylohyoid MEPs (>100 μV) with weak (5–10% of maximal) jaw contraction maintenance.

### 2.3. Magnetic stimulation of the motor cortex and MEP recording in patients with ICH

To assess swallowing function in patients with ICH, we validated the affected and unaffected motor cortex under NBS assistance to determine appropriate stimulus intensities for the RMT before and after QBS5 conditioning at the left motor cortex. The MEPs were measured from the affected and contralateral mylohyoid muscles, respectively. Unstable recording sessions (under 50 μV MEPs, peak-to-peak) after 30 trials due to severe swallowing dysfunction were interrupted.

### 2.4. Data analysis

Motor evoked potential data were analyzed offline as previously described (Sykes et al., 2016; Fujiki et al., 2021, 2022). All data are presented as the mean ± standard deviation (SD), and the level of statistical significance was set at *P* ≤ 0.05. For multiple comparisons among the different configurations used for stimulation, the latencies, RMT and MEP amplitudes were analyzed via two-way, repeated measures analysis of variance (ANOVAs). In these analyses, stimulus condition [SP, before and after QBS5 in both side mylohyoid MEP recordings] was used as a between-subject factor, while MWST scores [5–1] were used as a within-subject factor. *Post hoc* Bonferroni corrections was also employed in cases of multiple comparison. Correlations of post-ICH swallowing function with MEP parameters, RMTs, and amplitude were evaluated using correlation coefficients (r) and coefficients of determination (R^2^). The analyses were performed using SPSS (Cary, NC, USA).

## 3. Results

### 3.1. Mylohyoid MEP amplification after QBS5 in healthy controls

All participants did not report any adverse effects during or after the recording. Additionally, EEG abnormalities were not noted during or ≥30 min post-recording. The distance between the mylohyoid and FDI cortical representation was 23.22 ± 7.34 mm, with a range of 14.28–33.01 (Figure 2A).

Figure 2B illustrates representative right mylohyoid MEP traces after left hemisphere stimulation. The average traces for right and left mylohyoid muscle MEP amplification after left hemisphere QBS5 stimulation are shown in Figure 2C (Rt: pink, Lt: red). The gray boxes within the dashed represent each QBS5 burst. Time course analysis indicated that MEP amplification persisted for approx. 90-min after QBS5. The dark blue dashed box highlights the 30 min-left hemisphere-conditioning stimulus time window corresponding to Figure 1A). Immediately after QBS5 conditioning, mylohyoid muscle MEP amplitudes were higher than those at baseline, gradually decreasing to near-control levels by 90 min. Note that mylohyoid MEPs recorded from the bilateral side facilitated following QBS5-left hemisphere-conditioning. As shown in Figures 2A, B, the ANOVA revealed a significant main effect of MEP after QBS5, suggesting that the stimulation elicited different effects in the four-time points groups [main effect of GROUP, *F*(3,28) = 18.16, *P* < 0.001; main effect of TIME, *F*(5,118) = 12.75, *P* < 0.001; GROUP × TIME interaction, *F*(15,336) = 2.91, *P* < 0.001].

*Post hoc* analysis indicated that MEP amplitudes increased significantly relative to baseline and following SP-MEP after QBS5 left hemisphere stimulation in the Rt. and Lt. mylohyoid-MEPs (*P* < 0.001). Multiple comparisons between the QBS5 and baseline groups indicated that MEP amplitudes were increased for both Rt. and Lt. MEPs only immediately after QBS5 conditioning (*P* < 0.001, Figure 2C).

### 3.2. Characteristic RMT and amplitude profiles for mylohyoid MEPs in healthy controls

**FIGURE 3:**
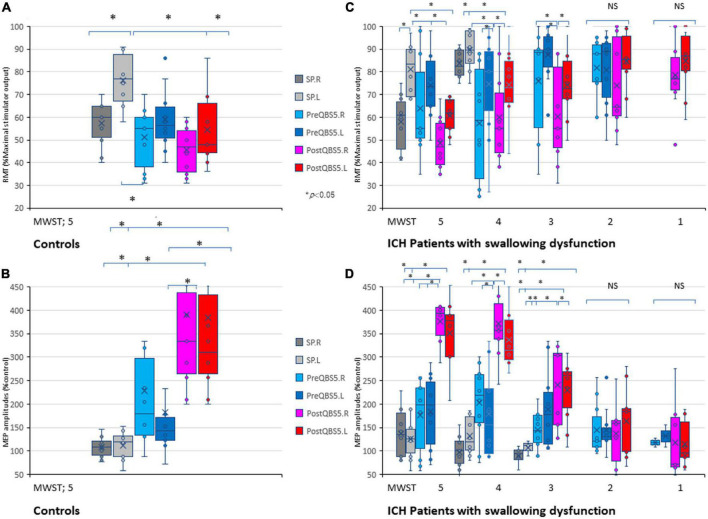
Validations of mylohyoid MEP parameters before and after QBS5 conditioning: comparison of characteristic RMT and amplitude profiles and the correlation with MWST scores in healthy controls and ICH patients. Quantitative differences in RMT **(A)** and amplitude **(B)** between the six-mylohyoid muscle conditions were statistically significant in healthy controls (*P* < 0.05). RMTs were significantly lower on the right side (*P* < 0.05). A multiple comparison test revealed significant differences in RMT and MEP amplitudes, and that the QBS5 induces higher amplitudes with lower stimulus intensities in healthy controls (*P* < 0.05). Quantitative differences in RMT **(C)** and amplitude **(D)** between the six-mylohyoid muscle conditions were compared in patients with ICH. Significant differences in RMT and amplitude (MWST scores 5 to 3) were observed in patients with ICH (**C,D**; *P* < 0.001). MWST scores were significantly correlated with RMTs and postQBS5-Rt./Lt.-MEP amplitudes (*P* < 0.001, respectively) but not those of preQBS5-Rt./Lt. or single-pulse-Rt./Lt. Colors in the graph represent each condition before and after TMS [red: postQBS5-Lt., pink: postQBS5-Rt.; dark blue: preQBS5-Lt.; light blue: preQBS5-Rt.; dark gray: single-pulse Rt.; right gray: single-pulse-Lt. in healthy controls **(A,B)** and MWST-parameter correlations in patients with ICH **(C,D)**, respectively]. QBS5, five-ms-quadri-burst stimulation; QPS, quadripulse stimulation; SP, single pulse; ICH, intracerebral hemorrhage; RMT, resting motor threshold; MEP, motor evoked potential; MWST, modified water swallowing test.

We compared SP-, 500Hz-QPS before and after left hemisphere QBS5 conditioned-MEPs in healthy controls to validate the methodological and physiological configurations used for each condition. Figures 3A, B and Table 1 show the comparison of RMTs, amplitudes before and after QBS5 and single-pulse MEPs. The ANOVA indicated that MEP parameters differed significantly among the bilateral six stimulation conditions (RMT: *F*(5,54) = 6.65 *P* < 0.001, amplitude: *F*(5,54) = 8.07, respectively, *P* < 0.001; Table 1). However, there were no significant differences in latency among the six conditions [*F*(5,54) = 0.364, *P* = 0.871]. In the *post hoc* analysis, both RMT and MEP amplitude differed among the conditions (*P* < 0.05; Figures 3A, B). Furthermore, we observed no significant differences in RMTs and latencies of SP-MEP parameters relative to baseline after QBS5 trials (RMT: 57.3 ± 10.34 vs. 57.4 ± 10.47%; latency: 12.4 ± 1.62 vs. 11.6 ± 3.78 ms; after SP-control and QBS, respectively). In addition, RMT [*t*(18) = −0.02; *P* > 0.05], latency [*t*(18) = 0.62; *P* > 0.05] were not significantly affected by QBS5.

When SP, before and after QBS5 conditioning MEP parameters for healthy controls were compared between hemisphere, only the right side mylohyoid MEP exhibited significantly lower RMTs for SP-MEP [RMT-Rt vs Lt, 57.3 ± 10.34 vs 76.4 ± 11.65; *P* = 0.008]. In addition, there were no significant differences in RMT or amplitude between right and left MEPs for other modalities [*t*(9) = 0.06 – 0.79 range; *P* > 0.05].

### 3.3. Correlation between MWST scores and mylohyoid MEP parameters in patients with swallowing dysfunction after ICH

In the ICH group, age (68.8 ± 7.6 years) and hematoma volume (5–28 ml, with a mean of 11.2 ± 4.87) were not significantly correlated with MWST scores. SP-mylohyiod MEPs were recordable only in patients with ICH with MWST scores 5 to 3 [5; 100%, 4; 100% and 3; 25(left)–33.3(right)%, respectively, Table 2].

**TABLE 2:** Quantitative differences in the mylohyoid MEP parameters between six conditions.

		Single		PreQBS5 conditioning		PostQBS5 conditioning		*F*	*P*
**MWST**		**SP-Rt.**	**SP-Lt.**	**PreQBS5-Rt.**	**PreQBS5-Lt.**	**PostQBS5-Rt.**	**PostQBS-Lt.**		
**Healthy controls**									
5	RMT (%)	57.3 ± 10.34	76.4 ± 11.65	51.9 ± 13.69	59.1 ± 14.12	45.5 ± 10.76	54.4 ± 15.96	*F*(5,54) = 6.65	**<0.001**
(*n* = 10)	Amplitude (μV)	108.1 ± 22.57	111.3 ± 30.68	228.8 ± 141.11	182.3 ± 133.7	390.6 ± 206.2	383.9 ± 208.21	*F*(5,54) = 8.07	**<0.001**
	MEP recordable	10	10	10	10	10	10		
**ICH patients**									
5	RMT (%)	57.9 ± 12.28	81.1 ± 11.45	63.7 ± 22.81	73.9 ± 14.48	48.5 ± 11.47	60.6 ± 7.86	*F*(5,54) = 6.76	**<0.001**
(*n* = 10)	Amplitude (μV)	136.8 ± 52.94	125.7 ± 42.86	176.4 ± 78.82	183.6 ± 79.09	375.6 ± 42.25	350.9 ± 71.86	*F*(5,54) = 30.07	**<0.001**
	MEP recordable	10	10	10	10	10	10		
4	RMT (%)	83.6 ± 6.12	89. ± 8.26	57.4 ± 25.81	74.5 ± 22.84	60.4 ± 21.23	74.4 ± 16.52	*F*(5,54) = 4.88	**<0.001**
(*n* = 10)	Amplitude (μV)	97.6 ± 37.01	132.2 ± 40.79	203.7 ± 79.25	179.3 ± 93.99	371.6 ± 77.52	336.1 ± 57.67	*F*(5,54) = 26.59	**<0.001**
	MEP recordable	10	10	10	10	10	10		
3	RMT (%)	100	100	75.9 ± 23.25	87.6 ± 12.06	60.4 ± 21.23	74.3 ± 14.78	*F*(5,49) = 5.78	**<0.001**
(*n* = 12)	Amplitude (μV)	89.5 ± 21.63	107.3 ± 15.82	144.2 ± 41.12	188.8 ± 85.62	226.9 ± 94.58	229.9 ± 66.93	*F*(5,49) = 4.37	**<0.001**
	MEP recordable	4 (33.3%)	3 (25%)	12	12	12	12		
2	RMT (%)	>100	>100	81.9 ± 13.37	80.9 ± 16.54	74.4 ± 20.27	84.8 ± 13.28	*F*(5,38) = 0.71	0.623
(*n* = 11)	Amplitude (μV)	Not recordable	Not recordable	108.4 ± 13.12	116.2 ± 25.44	137.1 ± 63.89	163.7 ± 73.49	*F*(5,38) = 0.27	0.924
	MEP recordable	0	0	11	11	11	11		
1	RMT (%)	>100	>100	100	100	78.4 ± 15.46	84.8 ± 13.81	*F*(5,22) = 1.48	0.23
(*n* = 12)	Amplitude (μV)	Not recordable	Not recordable	118.5 ± 14.14	131.5 ± 33.23	117.7 ± 74.98	114.9 ± 49.38	*F*(5,22) = 0.07	0.98
	MEP recordable	0	0	2 (16.7%)	2 (16.7%)	12	12		

MEP, motor evoked potential; RMT, resting motor threshold; QBS5, quadr burst stimulation-5ms interburst interval; QPS, quadripulse stimulation; SP, single pulse. Bold indicates *P*-value less than 0.05.

When the six mylohyoid MEP conditions (SP-Rt./Lt., PreQBS5-Rt./Lt., and PostQBS5-Rt./Lt.) were compared in each MWST score group, RMT and amplitude significantly differed for patients with MWST scores of 5–3 (*P* < 0.001; Table 1). A one-way ANOVA revealed that RMT and amplitude differed significantly among the six mylohyoid MEP conditions (RMT: *F*_(31,305)_ = 6.78 *P* < 0.001, amplitude: *F*_(31,305)_ = 11.45, respectively, *P* < 0.001). In the two-way repeated-measures ANOVA, the interaction between CONDITION (SP-Rt./Lt., PreQBS5-Rt./Lt., and PostQBS5-Rt./Lt.) and MWST (RMT: *F*(21,305) = 2.37, *P* < 0.001, amplitude: *F*(21,305) = 2.41, *P* < 0.001, respectively) was significant. The *post hoc* analysis also revealed significant differences in RMT (between PostQBS5-Rt. and PostQBS5-Lt.; PostQBS5-Lt. and PreQBS5-Rt., SP-Lt., respectively) and amplitude (between PostQBS5-Lt. and PreQBS5-Rt./Lt., SP-Rt./Lt., respectively, *P* < 0.001; Figures 3C, D).

We observed significant correlations among swallowing function (MWST scores), RMTs, and PostQBS5-MEP amplitude (RMT: *r* = −0.48, *R*^2^ = 0.23, *P* < 0.001/*r* = −0.46, *R*^2^ = 0.21, *P* < 0.001; amplitudes: *r* = 0.83, *R*^2^ = 0.68, *P* < 0.001/*r* = 0.83, *R*^2^ = 0.68, *P* < 0.001, PostQBS5-Rt./Lt. side, respectively; Figures 3C, D; pink [Rt.] and red [Lt.]). However, no such findings were observed for PreQBS5-Rt./Lt or single-pulse-Rt./Lt.-MEPs.

## 4. Discussion

The current findings indicate that mylohyoid-MEPs after left motor cortical QBS5 (four sets of four monophasic pulses at frequency of 500 Hz, repeated at 200 Hz; i.e., 5-ms interburst interval, with an inter-train interval of 5 s) conditioning, strongly facilitates bilateral mylohyoid MEPs. Previous studies have reported that changes in the excitability of cortical projections in various swallowing muscles can be observed using TMS-induced MEP (Doeltgen et al., 2011; Macrae et al., 2014). These studies provide valuable insight into the central nervous system response to dysphagia and, more importantly, to the adaptations associated with functional recovery. However, rigorous methodological controls and qualitative assessment measures are needed to obtain robust and clinically applicable findings in neurophysiological studies of swallowing.

### 4.1. Mapping of swallowing-related regions for stable, non-invasive evaluations of swallowing function

One of the gold standards for functional brain mapping is functional MRI (fMRI), which has been used to assess swallowing function for some time (Hamdy et al., 1999; Mosier and Bereznaya, 2001). Since Amassian et al. (1987) first recorded laryngeal MEPs using TMS, various efforts have been made to evaluate the function of the pharyngeal and laryngeal regions (Ertekin et al., 2001; Gallas et al., 2007; Khedr et al., 2008). These findings indicate that impairments in the cortical projections to swallowing musculature directly affect the sensitivity of MEPs. Impaired corticobulbar projections in patients may result in the disappearance of MEPs. On the other hand, high variability has been observed when measuring MEPs from the swallowing muscles, with research indicating that 40–50% of MEPs from the submental muscles cannot be recorded during volitional and reflexive swallows (Doeltgen et al., 2011).

Recent studies have also attempted to achieve swallowing related target in a multimodal fashion by integrating fMRI and MEP recordings for the lip orbicularis oris muscle (Li et al., 2020). Cricothyroid muscle-targeting for Broca’s area mapping (Rogiæ et al., 2014) is a highly sensitive and specific recording method that uses intramuscular wire electrodes. On the other hand, it is somewhat invasive, and there is room for improvement. The mylohyoid muscle sites for eliciting MEPs during swallowing assessments can be accurately determined by combining stable MEP and NBS mapping. Present result confirmed right side dominant laterization and bilateral facilitation after neuromodulations in the swallowing motor cortex mylohyoid muscle, only in RMT but not in latencies and amplitudes in comparison with previous research (Tang et al., 2022). Different stimulus configurations in the present study may affect the difference. In this regard, bilateral mylohyoid MEP facilitation after left side motor cortical QBS5 conditioning provides further important questions for swallowing neurophysiology and non-invasive neuromodulations. Original QPS5 induces LTP-like plasticity effects in the contralateral motor cortex in the healthy subjects (Tsutsumi et al., 2014). For further exploration, because the mylohyoid MEPs recorded in this study were less specific due to the compound muscle action potentials of the genio-mylo-dygastric-hyoideus muscles regions, and simultaneous bilateral-multi-muscle targeted recording is an issue for further investigation.

### 4.2. RMT and amplitude of bilateral mylohyoid-MEPs after left motor cortical QBS5 conditioning as surrogate quantitative biomarkers for swallowing dysfunction after ICH

Single-pulse TMS of the hand motor cortex induces 2-ms periodical descending volleys, resulting in temporal summation that generates cortico-muscular MEPs under healthy conditions (Amassian et al., 1990; Di Lazzaro et al., 2003). This is not always guaranteed in patients with motor paresis. In fact, MEP induction rates after single-pulse stimulation are considerably reduced in patients with severe motor paresis (Rogiæ et al., 2014). This is because configurations of corticospinal D and I wave descending volleys that generate cortico-muscular MEPs after a single TMS pulse are easily affected by various pathological conditions, resulting in failure of temporal summation at spinal motor neurons (Amassian et al., 1990; Di Lazzaro et al., 2003). In addition, it is unclear whether descending volleys occur at the same cycle from the cerebral cortex to the spinal nerve nuclei involved in human swallowing function. Moreover, swallowing movements are established by the integrated function of many neural regions. The primary motor cortex is involved in the induction of swallowing; the supplementary motor cortex is involved in the inhibition of swallowing movements; the cingulate gyrus is involved in the initiation of voluntary swallowing, and the sensory cortex is involved in the somatosensation of the pharyngeo-larynx in the swallowing reflex. Furthermore, the insula acts in coordination with the cortex and solitary bundle nucleus, the cerebellum contributes to regulation of areas in the primary motor cortex and other regions, and the basal ganglia play a role in the elicitation and regulation of swallowing via thalamocortical association areas (Hamdy et al., 1999; Mosier and Bereznaya, 2001). Therefore, the mylohyoid-MEP reflects only a portion of cortico-lingual muscle group function (i.e., the early voluntary motor phase of all swallowing), which may not be the most important factor to consider in assessments of swallowing function. Nevertheless, it is noteworthy that left side motor cortical QBS5 conditioning elicit 380–390% of the bilateral control MEP amplitude at significantly lower stimulus intensities. Furthermore, there were significant correlations between the mylohyoid-MEPs after left motor cortical QBS5 conditioning and MWST scores. These correlations were higher for amplitude than for RMTs, and linear correlations were observed for mylohyoid-MEPs after QBS5 alone. Although these correlations were relatively weak, they are clearly compatible with previous QTS results for FDI-MEPs (Fujiki et al., 2022). Mylohyoid MEP facilitations after QBS5 conditioning and QTS amplification for FDI MEP relies on fundamentally different neuromodulations, this supports the hypothesis that presynaptic projections to pyramidal cells in the swallowing-related hyoid muscle group are less frequent than in the hand muscles and therefore less accessible to transsynaptic stimulation (Guggisberg et al., 2001). In contrast, it is important to note that bilateral mylohyoid MEP facilitation after left side motor cortical QBS5 conditioning attenuates according to the severity grade of swallowing dysfunction. Exploration of corticospinal and corticobulbar tract excitability using QBS5 neuromodulation will contribute to our understanding of the mechanisms underlying stroke and subsequent pathophysiological changes.

### 4.3. Limitations and future work

The current findings suggest that QBS5 conditioned-mylohyoid MEP assessments represent a reliable, non-invasive method for quantifying swallowing dysfunction. However, this study has several limitations. First, present study recruited only patients with ICH, which comprise a lower proportion of stroke patients compared to cerebral infarction. This is because for the purpose that all participants only exhibited targeted motor paresis and swallowing dysfunction with small volume so that morphologically cerebral cortical intact, which was therefore favorable for motor function-oriented MEP studies. Accordingly, comparison between ICH and ischemic stroke patients with same degree of swallowing dysfunction may provide useful information about neurophysiological and pathopysiological mechanisms and/or understanding of swallowing dysfunction. Second, the mylohyoid-MEP reflects only a portion of cortico-lingual muscle group function (i.e., the early voluntary motor phase of all swallowing), and this may not be representative of total swallowing function. Careful interpretation is required given that QBS5 conditioned-mylohyoid MEP parameters may differ from corticospinal FDI and MWST scores, which are both non-linear (Macrae et al., 2014). Furthermore, QBS5; fundamentally different paradigm from original QPS5, to address potential safety issues, additional studies are required to determine the precise relationships of neurobehavioral features with the results of electrophysiological, morphological, and molecular-level assessments. Language mapping (Rogiæ et al., 2014; Honda et al., 2021) or neuromodulation-oriented therapeutics (Carmel et al., 2010; Müller-Dahlhaus and Vlachos, 2013) using the QBS5 paradigm may also be possible.

## 5. Conclusion

The current results suggest that left motor cortical QBS5 conditioning strongly facilitates bilateral mylohyoid MEPs both in controls and patients with dysphagia. Our analysis also indicated that MWST scores were significantly correlated with RMTs and amplitudes for mylohyoid-MEPs after conditioning, suggesting that these two parameters can be used as surrogate quantitative biomarkers in the assessment of swallowing function.

## Data availability statement

The original contributions presented in this study are included in the article/supplementary material, further inquiries can be directed to the corresponding author.

## Ethics statement

The studies involving human participants were reviewed and approved by the Ethics Committee of the School of Medicine at Oita University. The patients/participants provided their written informed consent to participate in this study.

## Author contributions

MF and NH designed the research paradigm. MF and HF analyzed the data. MF, NH, WM, and HF wrote the manuscript. All authors performed the research and contributed to the article and approved the submitted version.
